# Policies for supporting caregivers of older adults with long-term care needs in EU countries: a systematic review

**DOI:** 10.1007/s10433-026-00907-y

**Published:** 2026-02-05

**Authors:** Eva Bei, Marco Albertini, Federico Toth

**Affiliations:** 1 Department of Political and Social Sciences, University of Bologna, Strada Maggiore, 45, 40125 Bologna, Italy; 2 Department of Education, Languages, Interculture, Literatures and Psychology (FORLILPSI), University of Florence, Via Di San Salvi 12, 50135 Florence, Italy

**Keywords:** Europe, Caregivers, Long-term care, Policies, Ageing

## Abstract

In the context of population ageing, increasing long-term care needs and constraints on welfare spending, informal caregivers assume a pivotal role as providers of long-term care. This systematic review synthesises reported data on the diverse support policies implemented across the European Union (EU) to assist them. Findings from 35 studies and reports published between 2011 and 2025 suggest that, despite similar demographic challenges, policies diverge significantly, with some EU countries to have established mechanisms beyond financial assistance to support carers, while others, particularly those located in Eastern and Southern EU, to offer less comprehensive support. While the frameworks of defamilialisation (Nordic), supported familialism (Continental), and familialism by default (Southern and Eastern Europe) provide a useful analytical lens to our findings, the review also identifies significant heterogeneities within welfare regimes and even within countries, often driven by decentralisation and regional disparities. Across all models, financial support via cash-for-care schemes and in-kind respite care are the most common instruments, though their generosity and comprehensiveness vary substantially. In contrast, policies such as training, counselling, and flexible work guarantees are less developed, with their availability and access, however, varying significantly across the EU. Furthermore, the review identifies critical gaps in the geographical coverage of existing literature, with certain Southern and Eastern EU countries being particularly understudied. The synthesised evidence provides key implications for policymaking, thoroughly mapping the implementation of diverse social care policies as reported in the international literature.

## Introduction

In the face of a global demographic shift characterised by an ageing population, addressing the needs of older and care-dependent adults has become a critical imperative. Fuelled by increased longevity and decreasing fertility, this transformation has created a substantial demand for long-term care (LTC) in high-income societies (Zaidi et al. 2017). This growing need for LTC is largely unmet by public and market services, making informal care, provided by family members or friends, essential to fill this gap. While caregiving can be rewarding, it may also lead to a high burden and a deterioration in well-being over time (Verbakel et al. 2014), while also impacting employment opportunities and career trajectories, costs that are disproportionately borne by women due to traditional gender roles in care (Eurostat 2024).

Against this background, it is crucial to examine policies supporting informal caregivers for older adults with LTC needs. While a substantial body of social policy research has explored long-term care, an updated systematic synthesis of policies aimed at supporting informal caregivers across the European Union (EU) is still needed. Informal caregivers have a range of multidimensional needs that extend well beyond financial assistance. Among others, effective policies must address work-caregiving reconciliation, provide training, offer emotional support, and deliver in-kind services to relieve families (Spasova et al. 2018). It is therefore essential for policymakers to implement a variety of tools to meet these diverse needs and support caregivers effectively.

Across different EU countries, LTC policies are shaped by national healthcare systems, welfare state institutions, prevalent family structures, cultural attitudes towards caregiving, and available resources. To support caregivers, EU nations utilise policy mixes that combine financial instruments, in-kind services, and regulation (Wieczorek et al. 2022). Financial benefits, including direct care allowances and indirect allowances for care recipients, are often the primary tool to mitigate the economic strain of caregiving. Indirect support, like tax relief and allowances for care or medical expenses, is also provided in some EU countries. Labour policies, including paid or unpaid leave and flexible work arrangements, aim to reconcile care with employment (Courtin et al. 2014; Wieczorek et al. 2022). In-kind support provides direct relief through respite care initiatives, while services like training and counselling help caregivers cope with the psychological burden (Spasova et al. 2018; Tokovska et al. 2022).

The composition and generosity of these policy mixes vary significantly across the EU, reflecting the complex social, cultural, and economic environments in which caregiving occurs (Wieczorek et al. 2022). To interpret such diversity, comparative welfare state theory provides a set of conceptual frameworks that have been widely applied to LTC and caregiving. Esping-Andersen’s seminal typology (1990) classified welfare regimes based on the balance between state, market, and family in welfare provision. His concepts of “decommodification”, the extent to which individuals can maintain a livelihood independent of the market, and “stratification", the reproduction of social inequalities, help explain why some countries rely on public provision, while others delegate more responsibility to families or the market.

Building on this foundation, and integrating the concept of “defamilialisation”—originally introduced by Lister (1994) and McLaughlin and Glendinning (1994) and later adopted by Esping-Andersen (1999)—scholars such as Leitner (2003) and Saraceno and Keck (2010, 2011) applied this lens directly to care. They distinguished specific types of “familialism” and “defamilialisation”. “Familialism” may be “familialism by default”, where no public alternatives are available to the care dyad; “prescribed familialism”, where legal obligations to provide/finance care are enforced; or “supported familialism”, where allowances and care leave schemes assist families and carers in their role of care provision. “Defamilialisation”, by contrast, may take the form of support primarily through the market, via cash-for-care benefits enabling users to purchase services, or public provision, through in-kind services such as home care services and institutional support. More recently, scholars have further refined these concepts. Le Bihan et al. (2019) revisited the concept of “optional familialism”—originally identified in Leitner’s (2003) typology—to describe policy logics that actively empower family care as a genuine choice alongside state or market services. Complementing this, Eggers et al. (2020) have re-conceptualised “defamilialisation” and “familialisation” not as opposites, but as distinct policy dimensions that can coexist and vary independently within national welfare systems.

Together, these contributions underline that care policies are not merely technical instruments but part of broader welfare state arrangements. They help explain why support varies across EU and why it is useful to examine not only the policies themselves but also how they are studied and interpreted in research. By reviewing the scholar literature, we can capture both dimensions: the policy content and the ways in which scholars have analysed and classified these measures across contexts, providing a foundation for understanding cross-national differences while ensuring methodological transparency.

Against this background, the present review systematically maps existing policies addressing (directly or indirectly) caregivers’ needs, as reported in academic and grey literature. Focusing on EU member states allows for an examination of diverse national systems within a shared context. The objective is not to catalogue regulations directly but to synthesise how policies have been described, classified, and evaluated in research. The review addresses the following key research questions:

1a. What types of policies have been implemented, according to the scientific literature, across different EU countries to support informal caregivers of older adults with LTC needs?

1b. How do these policies vary, according to the literature, across different welfare regimes and geographical regions within the EU?

Throughout the review, we adopt a schema that distinguishes the following types of measures: compensation (direct caregiver allowances and allowances for care recipients in the form of cash payments); labour market policies (paid care leave providing job-protected leave with income replacement; unpaid care leave providing job-protected leave without compensation; and flexible work arrangements including rights to adjust working hours, work part-time, or work from home to accommodate caretaking responsibilities); social security coverage (pension credits or contributions, health insurance, and unemployment benefits tied to caregiving periods); physical and mental well-being support (training and information services; respite care including temporary relief and LTC services such as day care centres, short-term residential care, and home-based respite; counselling or emotional support services). This typology reflects how caregiver support policies have been classified in existing comparative research (e.g. Courtin et al. 2014; Spasova et al. 2018; Wieczorek et al. 2022) including policies directly targeting caregivers as well as those for care recipients that provide indirect support.

## Design and methods

### Searches

A detailed search strategy, covering publications from 2011 to 2025, was conducted across three relevant electronic databases (Scopus, Web of Science, and PubMed) to search for studies on informal care policies. Grey literature, including the OpenGrey database, Google Scholar secondary searches, and the reference lists of all studies, was also screened.

The search strategy was developed using key terms related to the caregiving population and the primary outcome of interest. A variation of controlled vocabulary, relevant subject headings when possible and free-text terms contained in the title/abstracts of publications was applied. An example of the search strategy string used for the Scopus database is presented below:

(Europe OR EU OR Austria OR Belgium OR Bulgaria OR Croatia OR Cyprus OR Czech Republic OR Denmark OR Estonia OR Finland OR France OR Germany OR Greece OR Hungary OR Ireland OR Italy OR Latvia OR Lithuania OR Luxembourg OR Malta OR Netherlands OR Poland OR Portugal OR Romania OR Slovakia OR Slovenia OR Spain OR Sweden) AND (Policy OR policies) AND (caregiver AND support OR care AND support OR carer* AND support OR assistance OR financial OR legal OR cash) AND (relative OR informal OR kin OR family OR caregiver* OR informal AND caregiver* OR carer*).

The search strategy string was adapted to the other two electronic databases. Included studies were restricted to those written in English. In line with the review objectives, we focused on published studies/reports rather than conducting a direct analysis of policy documents or databases. This choice allows us to synthesise how care policies have been reported, classified, and assessed in the literature, thereby capturing both the diversity of measures and their interpretation/representation in research.

### Study selection

All citations were imported into Zotero and duplicates were removed. In the primary screening stage, one reviewer screened titles and then abstracts based on the inclusion criteria, with a second author consulted on the process. Following this, two reviewers completed a full-text review of all eligible or potentially relevant studies. A third author was consulted to resolve any discrepancies that arose during the full-text review.

### Strategy for data synthesis

The data synthesis followed the Joanna Briggs Institute (JBI) convergent integrated approach for mixed-methods reviews (Lizarondo et al. 2020). Quantitative data were transformed into textual descriptions and pooled with findings from qualitative studies/reports. The combined data were then analysed to identify categories based on similarity, culminating in a set of integrated findings.

## Findings

**Fig. 1:**
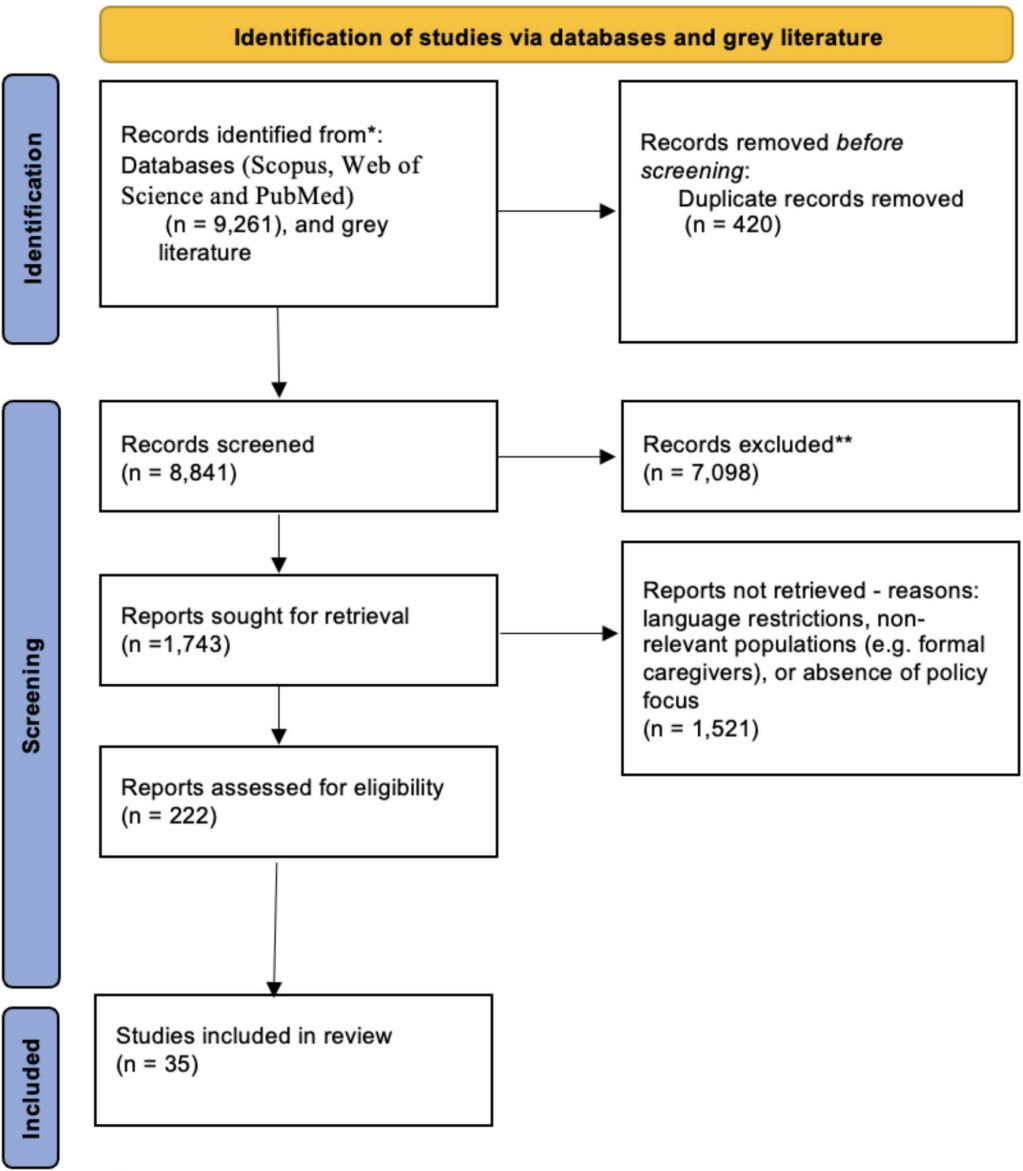
PRISMA flow diagram of study selection

A total of 35 studies were included in the review. Figure 1 summarises the selection process according to the PRISMA guidelines. Outside the selection process from database searches, additional policy reports and articles from grey literature, secondary searches on google scholar, and citation tracking, were also included.

### Characteristics of the Studies

Table 1 provides a detailed description of the findings from each reviewed study (study numbers listed in Table 1 are used to reference specific studies). The publication period spanned from 2011 to 2025, encompassing diverse methodologies including quantitative (Albuquerque 2022; Andersson et al. 2019; Arts 2002; Brega et al. 2023; Cahill et al. 2023; Calvó-Perxas et al. 2018; European Commission 2021; Le Bihan et al. 2019b; Lethin 2016; Lister 1994), qualitative studies (Calvó-Perxas et al. 2021; Esping-Andersen 1990; Llena-Nozal et al. 2025), and qualitative document analyses and reports (Barák 2022; Bouget 2016; Castles 2008; Courtin 2014; Di Rosa 2011; Eggers 2020; Eggers 2023; Esping-Andersen 1999; Eurofound 2025; Eurostat 2024; Ferrera 1996; Genet 2012; Jelley et al. 2021; Johansson et al. 2011; Kim et al. 2024; Laporte et al. 2018; Le Bihan 2012; Le Bihan 2019a; Le 2023; Leitner 2003; Lizarondo et al. 2020; McLaughlin 1994). Sample sizes varied widely, from small-scale qualitative interviews to large-scale surveys with thousands of respondents.

**Table 1:** Brief summary of studies in the systematic review (numbers are used in the Findings and Discussion sections to reference particular studies)

Study	Methods	Location	Sample Size (N)	Measures	Main Findings
1 Albuquerque 2022	Quantitative	Southern Europe: Portugal, Spain, Italy, Greece	N = 16,303 older adults aged 50 +	Support Policies: Cash provisions; in-kind provisions; recognition of informal carers and care allowances; system fragmentation	Portugal reported the highest proportion of older adults with care needs among the four countries, with Portuguese women particularly disadvantaged. Greece and Portugal showed higher probabilities of receiving exclusively informal care. Italy and Greece had the lowest probabilities of care involving formal services. Spain had the lowest probability of receiving exclusively informal care
2 Anderson et al., 2019	Quantitative	Sweden	N = 129 Working informal caregivers	Support Policies: Valued and received care of ICT and non-ICT support for caring	The results suggested a rather low level of received support including ICT and non-ICT support, for working carers. Low level of receipt of valued forms of support such as 1) access to information about caring issues of relevance to their own situation; 2) talking about their situation and share their experiences with others; 3) having practical assistance in caring; and 4) having access to relief and respite care
3 Barák et al. 2022	Quantitative	Czech Republic	N = 183 Informal caregivers	Support Policies: Introduction of the long-term carer’s allowance as a tool for supporting informal carers (and caregivers’ perceptions on the tool)	The introduction of the long-term carer’s allowance to support informal care was not considered a comprehensive benefit increasing the carers’ quality of life and motivation to care
4 Bouget et al. 2016 (European Commission)	EU report Comparative synthesis of national expert reports on social policy	Europe (35 countries, including all EU members at the time)	N = N/A	Support Policies: Work-life balance measures including care leave, flexible working arrangements, and care services for dependent family members	While most countries report care leave policies, their generosity, and conditions vary significantly. Long-term leave is frequently unpaid, and rights to flexible work are often limited to requesting changes rather than a guaranteed entitlement, creating a gap between policy existence and practical support for carers
5 Cahill et al. 2023	Qualitative Analysis of national policies	Sweden, Ireland, USA	N = N/A	Country Classification: analysis based on a nine-dimension framework to guide observations of policies and programmes of care in the three national contexts Support Policies: Type of long-term care regimes; legislation entitling caregivers to services; national dementia plan/strategy; national caregiver’s strategy/caregiver’s rights; support for working caregivers financial support, flexible work hours, paid leave; financial support for non-working caregivers	The Swedish model of welfare best reflects a universal care regime where Swedes pay high taxes but at least on paper, secure individual rights to support. Ireland was rated as a mixed solidarity/residual model, reflecting a lower level of support for caregivers in several types of services and programmes
6 Calvó-Perxas et al. 2018	Quantitative (Data from Wave 5 of SHARE)	EU (12 countries) Austria, Germany, Spain, France, Belgium, Czech Republic, Sweden, Netherlands, Denmark, Switzerland, Luxembourg, Slovenia	N = 13,507 Informal Caregivers	Country Classification: Family-based or Service-based (based on the welfare model, considering the role of family as informal caregivers and the level of professional services use) Support Policies: Aggregated into "Financial Support" (caregiver allowance, allowance for the person being cared for, tax credit, additional benefits, paid leave) and "Training and Other Types of Support" (unpaid leave, flexible work arrangements, training/education, respite care, counselling)	Non-financial support measures (education, training, respite care, non-paid leave, and counselling) had a larger protective impact on the health of caregivers than do financial support measures available in some European countries, regardless of the gender of the caregiver
7 Calvó-Perxas et al. 2021	Quantitative (Data from Waves 5 and 6 of SHARE)	EU (11 countries) Austria, Germany, Spain, France, Belgium, Czech Republic, Sweden, Denmark, Switzerland, Luxembourg, and Slovenia	N = 37,488	Country Classification: based on caregiving frequency: Scandinavian, Continental, Eastern, and Mediterranean countries Support Policies: caregiver allowances, allowances for the person being cared for; tax credits; additional benefits; paid leave, unpaid leave; flexible work arrangements, training/education; and respite care	Respite care and caregiver allowances emerged as the most effective measures of support for caregivers’ health maintenance. Countries that lacked support measures for informal caregivers were also those that showed the greatest risk of health decline
8 Courtin et al. 2014	Qualitative National experts interviews supplemented by literature review	Europe (23 countries) Austria, Belgium, Bulgaria, Czech Republic, Denmark, England, Finland, France, Germany, Greece, Hungary, Ireland, Italy, Latvia, Lithuania, Luxembourg, Malta, Netherlands, Portugal, Romania	N = 27 National experts	Support Services: care allowance; attendance allowance; counselling; information; respite care; training; pension credits; paid leave; unpaid leave	Financial support was found to be the most common type of assistance, followed by respite care and training. Most countries were reported to lack a systematic process to identify informal carers and assess their needs with the level of support varying significantly across the EU
9 Di Rosa et al. 2011	Quantitative	EU (6 countries) Greece, Italy, UK, Sweden, Poland, Germany	N = 5,923 Informal caregivers	Support Services: Support services were categorised into two main groups: services directly addressed to the older care recipient and services for the caregiver	The country-level data analysis shows that family carers of older people in Sweden, the UK, and Germany can count on a more developed network of specific support services including socioemotional support. Nevertheless, very few caregivers actually received these services. By comparison, these services are almost totally absent in Greece, Italy, and Poland, countries in which caregivers often find support from services not originally created to specifically address family carers’ needs, but that may act as a substitute for unavailable services
10 Eggers et al. 2020	Qualitative Comparative analysis of policy data	EU (5 countries) (Denmark, Germany, Czech Republic, Italy, Ireland)	N/A	Support Policies: Generosity of LTC policies on extra-familial care (e.g. home and residential care services) and paid family care (e.g. care allowances, social security rights for caregivers)	The study finds that de-familialising and familialising policies are not mutually exclusive and often coexist. Four country clusters are identified based on their LTC policy generosity
11 Eggers & Grages 2023	Qualitative Comparative analysis of care policy documents, standardised policy reports, and comparative databases	Europe (5 countries) Norway, Germany, England, Italy, Estonia	N/A	Support Policies: Comprehensive analysis of policy generosity across five indicators: access to public support, pay for family care, social security rights, work-related rights, and public support for extra-familial home care. Analysis framed by social risk theory and welfare regime typologies	Cross-national comparison reveals Norway and Germany show relatively high potential for alleviating family carers' social risks through "optional familialism". England, Italy, and Estonia show limited potential, with Estonia having particularly weak support and legal obligations for family care. Traditional welfare regime typologies only partially explain current care policy designs
12 Eurofound 2025	EU report Analysis of policy frameworks across Member States supplemented by survey data from ESS, CARE, and SHARE	All 27 EU countries	N/A	Support Policies:reconciliation measures; formal care support; information provision, education and training; social protection rights; income support; health and well-being interventions; young carer support	Significant heterogeneity in care policies across the EU. Financial support varies widely. Nordic countries provide a more comprehensive policy mix; Eastern and Southern Europe offer more limited support. Young carers remain largely invisible in policy frameworks across most Member States
13 European Commission, 2021	EU report Analysis of LTC policies	All 27 EU countries	N/A	Support Services: long-term care policies; counselling; training; respite care; cash benefits; paid leave; unpaid leave	Significant gaps are highlighted in accessing formal care and social protection across EU, as well as staff shortages and challenging working conditions despite the sector's job creation potential
14 Genet et al. 2012 (WHO)	WHO report Comparative project (systematic literature review, policy document analysis, statistical data, expert interviews)	31 European countries	N ≈ 10 experts per European country; supplemented with documents and secondary data	Indicators on governance, financing, service delivery, human resources, quality, and role of informal carers	Documented large cross-national and regional variation in home care organisation, financing and access across Europe. Highlighted the interdependence between professional services and informal care, and the reliance of many systems on family carers. Found strong regional inequalities in decentralised systems
15 Jelley et al. 2021	Qualitative Semi-structured interviews with content analysis	Europe (8 countries) Netherlands, UK, Germany, Sweden, Norway, Ireland, Portugal, Italy	N = 390 Caregivers providing care to an older adult with Dementia	Support Services: support services for caregivers and care recipients; day care centres; and respite care	Majority of the support services were accessed to provide personal care at home or to meet physical needs. Only a limited number of services were used for companionship or social activities at home for the care recipient
16 Johansson et al. 2011	Qualitative Policy analysis of legislative texts, preparatory papers, committee reports, and government bills	Sweden	N/A	Support Services: Legislative developments regarding informal caregiver support analysed across three dimensions: 1) Support for physical and mental well-being; 2) Recognition and compensation; 3) Services and legal framework	Sweden's 2009 Social Services Act amendment changed municipalities' responsibility, granting the right to needs assessment. The amendment extended coverage to all caregiver groups. Implementation varied widely across municipalities due to local autonomy. The law did not specify the content or quality of support to be provided
17 Kim et al., 2025	Qualitative Comparative case study (analysis of policy documents, government reports, and legislation)	South Korea, UK, Sweden	N/A	Support Services: Three categories analysed: 1) Improving physical and mental well-being; 2) Compensation and recognition of informal caregivers; 3) Support for reconciling employment and informal care	South Korea views caregivers as resources and co-clients with limited well-being support and strict financial aid. The UK assigns co-worker and co-client roles with broader financial assistance. Sweden prioritises formal care and recognises caregivers as co-clients, placing strongest emphasis on well-being support through needs assessments, direct services, and coordinated respite care, while regulating financial aid and limiting employment-care reconciliation to end-of-life situations with paid leave at ~ 80% income for up to 100 days
18 Laporte et al., 2018	Quantitative	Germany	N = 560 13 Dementia Care Networks (DCNs)	Support Services: Home care nursing services; day care and short-term care; low-threshold services	Home care nursing services, day care, and short-term care were the most utilised sources of formal support. Relatively low and even decreasing utilisation rates of low-threshold services and companion home services were reported
19 Le Bihan & Martin 2012	Qualitative Policy documents analysis and semi-structured interviews with family caregivers	EU (4 countries) France, Italy, the Netherlands, Portugal	N = 86 Working informal caregivers	Support Policies: Analysis of policy trajectories and identification of policy measures in the four national contexts	Analysis of the interaction between policy measures and care practices at family level has shown a process of diversification in the four countries, through the development of various schemes including cash allowances, care leaves, and care services
20 Le Bihan et al., 2019a	Qualitative Comparative synthesis of national expert reports on social policy	Europe (12 countries) Austria, Bulgaria, Czech Republic, England, Finland, France, Germany, Italy, Latvia, Poland, Spain, Sweden	N/A	Support Policies: Three main types of direct support measures: 1) Compensation measures; 2) Supportive measures; 3) Conciliation/reconciliation measures	Indirect compensation via care recipients is more common. Supportive measures are largely absent in Bulgaria, Latvia, and Poland, but widely available in Austria, England, Finland, France, Germany, and Sweden. Conciliation measures are absent in Latvia, Poland, but varied in Austria, Germany, Italy
21 Le Bihan et al., 2019b	Qualitative Comparative analysis of policy documents	Europe (7 countries) Austria, Germany, France, Italy, Spain, Netherlands, England | N/A (policy analysis covering trajectories from early 1990s to mid-2010s)	N/A	Support Policies: Analysis of cash-for-care schemes within broader LTC policies and their relationship to informal care, examining policy trajectories over 20 years. Three types of measures examined: 1) Compensation measures; 2) Supportive measures; 3) Conciliation measures	A general turn towards “optional familialism” through the market observed across countries, where informal care is fostered, but families are also given some alternatives. All countries showed increasing recognition of caregivers through legislation and expansion of support measures including paid leaves, social security coverage, and training
22 Lethin et al. 2016	Qualitative Document analysis	Europe (8 countries) Estonia, England, Finland, France, Germany, the Netherlands, Spain, Sweden	N/A	Support Policies: A mapping system was used in 2010–2011 to gather information about estimations of availability, utilisation, and providers of support for informal caregivers of older people with Dementia	Counselling, caregiver support, and caregiver education were the support activities with high availability from diagnosis stage to the intermediate stage, with a decrease in the late to end-of-life stage. Utilisation was low, with a small increase in the intermediate stage. Day care and respite care at home had the highest availability from the diagnostic to the intermediate stage, with a decrease in the late to end-of-life stage
23 Llena-Nozal et al. 2025 (OECD)	OECD report Comparative analysis using clustering methodology based on quantitative and qualitative policy data	OECD countries	N/A	Support Policies:LTC system classification across five dimensions including: availability and support for informal carers; coverage of formal LTC services; public funding generosity; means-testing and needs-testing; governance and integration	Four LTC system clusters identified based on comprehensive analysis. Support for informal carers varies substantially: Cluster 1 (Nordic and Continental Europe) provides at least two policy measures and highest public funding; Cluster 2 (mixed systems) offers high formal care with informal carer support; Cluster 3 (decentralised systems) has limited caregiver support (1–2 policies); Cluster 4 (Southern and Eastern Europe) shows highest informal care prevalence but minimal policy support (0–1 policies), with lowest public funding
24 Pavolini 2021 (ESPN)	ESPN report Comparative synthesis of policy data from ESPN country reports	EU (27 countries)	N/A	Support Policies:Organisation of LTC systems; types of social protection provision; eligibility criteria; financing mechanisms; coverage rates; out-of-pocket expenditure	Six LTC models identified based on public expenditure level and share allocated to cash benefits: Limited state intervention model includes Southern and Central-Eastern European countries; Mild intervention through cash benefits and mild intervention through services models show moderate spending; Strong intervention through cash benefits model combines services and generous cash transfers; Strong intervention through services model features integrated systems with high service provision; Very strong intervention through services model includes Nordic countries with comprehensive universal coverage primarily through formal services
25 Ranci et al. 2019	Qualitative Comparative analysis of policy documents, official papers, and grey literature	Europe (6 countries) Austria, Germany, France, Great Britain, Italy, Spain	N/A	Support Policies: Cash-for-care programmes examining eligibility criteria, need assessment procedures, classification of disability levels, and benefit amounts across different dependency levels	Cash-for-care programmes differ substantially in coverage and generosity. Austria has the most inclusive and generous system. Germany is more selective but generous, with standardised assessment and progressive benefits targeting those most in need. Italy and Great Britain have very extensive coverage but lack progressivity, with flat-rate or two-level benefits that fail to provide adequate support for severe disabilities. France and Spain are more restrictive in access but provide progressive support
26 Riedel et al. 2016	Qualitative Comparative analysis of policy documents	EU (21 countries) Austria, Belgium, Bulgaria, Czech Republic, Denmark, England, Estonia, Finland, France, Germany, Hungary, Italy, Latvia, Lithuania, Netherlands, Poland, Romania, Slovakia, Slovenia, Spain, Sweden)	N/A	Support Policies: Organisation and supply of LTC systems (governance, financing, access, service mix of institutional and home care, role of public/private actors)	Wide variation across Europe in organisation and financing of LTC. Most countries provide needs-based entitlements but require user cost-sharing; governance arrangements differ between centralised and decentralised systems. Regional disparities in access and waiting times are common
27 Rocard & Llena-Nozal 2022	OECD report Analysis of policies to support caregivers	OECD countries	N/A	Support Services: counselling; training, respite care; cash benefits; paid leave; unpaid leave	About two-thirds of countries provide cash benefits to informal carers, either directly or through care recipients. One-third of countries lack social security coverage tied to cash benefits. There is a growing commitment to support working carers, with many countries offering paid leave, but flexible work arrangements for caregivers are still uncommon
28 Rogero-García, 2016	Quantitative (Survey on the use of caregiver leaves)	Spain	N = 896 Working caregivers	Support Services: paid care leave	Care leave is not often used, especially compared to leave-taking to care for children. Just 3.2% of the caregivers who had cared for dependent adults in the last 20 years reported having reduced their working hours or taken a full leave for that purpose
29. Spasova et al. (2018)	ESPN Report	Europe (35 countries) Belgium, Denmark, Czech Republic, Germany, Estonia, Ireland, Cyprus, Greece, Latvia, Lithuania, Hungary, Malta, Poland, Slovenia, Slovakia, Portugal, Finland, Sweden, Bulgaria, Romania, Croatia, Former Yugoslav Republic of Macedonia, Iceland, Liechtenstein, Norway, Serbia, Switzerland, Turkey	N/A	Support Services: cash benefits; formal care services	National LTC arrangements for the elderly (65 +) vary significantly across Europe in terms of organisation, funding, and types of care offered. Common challenges include fragmented access and financing, a trend towards prioritising home care despite underdevelopment, and the prevalence of informal care due to limited formal LTC options
30. Tokovska et al., (2022)	Qualitative document analysis of15 national dementia strategies from 2012 until 2030	Europe (15 countries) Austria, Belgium, Czech Republic, Denmark, Finland, Germany, Gibraltar, Ireland, Israel, Malta, Netherlands, Norway, Sweden, the UK (Scotland and Wales)	N/A	Support Policies: Document analysis of 1) psychosocial support EU policies, and 2) implementation of technologies to enable psychosocial support of caregivers	Psychosocial support is provided in various modalities: Education and training for informal caregivers through support groups; peer groups; consultations; counselling; care coordination; self-help meetings; exchange of experiences with dementia; public campaigns. Only some countries (e.g. Belgium, Denmark, Finland, Ireland) have flexibly moved psychosocial support to the online space
31. Verbakel (2018)	Quantitative (European Social Survey Round 7)	Europe (19 countries) Austria, Belgium, Czech Republic, Denmark, Estonia, Finland, France, Germany, Ireland, Lithuania, Netherlands, Norway, Poland, Portugal, Slovenia, Spain, Sweden, Switzerland, UK	N = 32,894 Informal caregivers	Country Classification: Nordic, South, East, Anglo-Saxon countries Support Policies: Formal LTC Provisions as measured by an index combining four indicators including: long-term care beds; long-term care workers; long-term care public expenditure; and the proportion of the population receiving long-term care	The Nordic countries had relatively many caregivers, but few intensive caregivers. A mixed group of countries in South, East, and Anglo-Saxon Europe had relatively few caregivers, but many intensive caregivers. Generous LTC provisions in a country were related to a higher likelihood of providing informal care, but a lower likelihood of providing intensive care
32. Verbakel et al. (2023)	Quantitative Indicator construction using secondary macro-level data	OECD countries	N/A (macro-level data)	Macro-level indicators of supportive LTC policy types: *Supported familialism*—caregiver support index; *Supported defamilialisation through the market*—cash benefits to care recipients; *defamilialisation through public provision*—LTC beds per 1,000 population aged 65 +	Descriptive mapping showed higher caregiver support index in Scandinavia and parts of Western Europe; caregiver cash benefits more common in Eastern countries and Scandinavia; cash benefits to care recipients present in ~ 75% of observed OECD countries; higher public provision in Switzerland, Sweden, the Netherlands, and Luxembourg with lower levels in Eastern and Southern Europe
33. Wieczorek et al., (2022)	Qualitative Commentary analysis of national policy strategies within EU	EU countries	N/A	Support Policies: carer compensation and recognition; labour market policy; and carers’ physical and mental well-being	Cash benefits are a particularly common method of supporting informal caregivers; paid and unpaid leave, and flexible work arrangements are the most prevalent measures to support family caregivers within labour market policy
34. Willemse et al. (2016)	Qualitative Semi-structured interviews	EU (5 countries) Belgium, The Netherlands, Luxembourg, France, and Germany	N = 10 (5 caregivers and their care recipient)	Support Policies: formal services for the dependent elderly; support measures for the informal caregiver	Formal services such as home care were reported to have the largest impact on allowing the caregiver to care for the dependent elderly at home. Policy measures, such as respite care and psychosocial support, were seldom used or known. Financial compensation services, were not always provided in the city/village of some of the respondents
35. Zigante (2018) (European Commission)	EU report Scoping study of literature, policy documents, and legislation	Europe (7 countries) Czech Republic, England, France, Germany, Netherlands, Spain, Sweden	N/A	Support Policies: Exploration of “formalisation” of informal care through three main facets: 1) Cash-for-care schemes and cash benefits; 2) Training and validation of carers' skills; 3) Carers' assessments and legislation recognising status and rights	More regulated schemes (UK, Netherlands, France, Sweden) offer protection for informal carers. Strictly regulated cash benefits encourage formal market and complementary formal care services, while unconditional cash benefits may create “incentive traps” encouraging carers to leave labour market. Training opportunities vary significantly with voluntary sector often playing large role

*ESPN* European commission and the European social policy network (ESPN), *ICT* information communication technology, *LTC* long-term care, *N/A* = Not Applicable; OECD = Organisation for Economic Co-operation and Development. SHARE = Survey of Health, Ageing and Retirement in Europe

The studies covered a broad geographical range, though the literature is unevenly distributed. While countries like Germany, France, and Spain have been extensively studied, others, particularly in Southern (e.g. Cyprus, Greece, Malta) and Eastern Europe (e.g. Bulgaria, Romania, Latvia, Lithuania), are relatively neglected. Key themes included the availability of support policies, the impact of LTC provisions, and the utilisation of specific services targeting the well-being of the caregiving dyad. In some instances, studies reported differing information for the same country, potentially due to outdated data or a focus on regional versus national policies. In cases of discrepancy, we prioritised the most recent national-level data.

### Integrated Synthesis of Findings

This integrated synthesis addresses the review's research questions by summarising the key policies implemented across EU countries to support informal caregivers, as reported in the literature. The findings are synthesised by region, highlighting how support mechanisms differ across various welfare regimes and geographical areas. Table 2 maps these support policies as they emerge from the relevant literature.

**Table 2:** Support policy measures for informal caregivers across the EU

EU Country	Compensation		Labour market policy			Social security coverage (pension and/or health and/or unemployment	Improving caregiver’s physical and mental well-being			Papers examined support policies per country
	Allowances for the person being cared for	Caregiver allowance	Paid leave	Unpaid leave	Flexible work arrangements		Training services and/or information	respite care	Counselling services/emotional support	
*Nordic Europe*										
Denmark	**Yes** (possibility to hire spouses)	**Yes**	**Yes**	**No**	N/A	Social insurance coverage	**Yes**	**Yes**	**Yes**	4, 6, 7, 8, 10, 12, 13, 14, 23, 24, 26, 27, 29, 30, 31, 32, 33
Finland	**Yes**	**Yes**	**Yes**	**Yes**	N/A	Social insurance coverage/ tax credits/reductions	**Yes**	**Yes**	**Yes**	4, 8, 12, 13, 14, 20, 22, 23, 24, 26, 27, 29, 30, 31, 32, 33
Sweden	**Yes**	**Yes**	**Yes**	**No**	N/A	Social insurance coverage	**Yes**	**Yes**	**Yes**	2, 4, 5, 6, 7, 9, 12, 13, 14, 15, 16, 17, 20, 22, 23, 24, 26, 27, 29, 30, 31, 32, 33, 35
*Continental Europe*										
Austria	**Yes**	**No**	**Yes**	**Yes**	**Yes**	Financing for respite care; social insurance coverage	**Yes**	**yes**	**Yes**	4, 6, 7, 8, 12, 13, 14, 20, 21, 23, 24, 25, 26, 27, 30, 31, 32, 33
Belgium	**Yes**	**Yes**	**Yes**	**Yes**	**Yes**	Tax reductions; tax credits applied to service vouchers to employ home workers	**Yes**	**Yes**	**Yes**	4, 6, 7, 8, 12, 13, 14, 23, 24, 26, 27, 29, 30, 31, 32, 33, 34
France	**Yes** (possibility to choose formal or informal care providers with the exception of spouses)	**No**	**Yes**	N/A	**Yes**	Carer credits/tax reliefs/ tax credits applied to service vouchers to employ home workers	**Yes**	**Yes**	**Yes**	4, 6, 7, 8, 12, 13, 14, 19, 20, 21, 22, 23, 24, 25, 26, 27, 31, 32, 33, 34, 35
Germany	**Yes** (possibility to choose formal or informal care providers)	**No**	**Yes**	**Yes**	**Yes**	Social insurance coverage/tax credits	**Yes**	**Yes**	**Yes**	4, 6, 7, 8, 9, 10, 11, 12, 13, 14, 15, 18, 20, 21, 22, 23, 24, 25, 26, 27, 29, 30, 31, 32, 33, 34, 35
Luxemburg	**Yes**	**No**	**Yes**	**Yes**	N/A	Social insurance coverage/tax reductions for LTC services	**Yes**	**Yes**	**Yes**	4, 6, 7, 8, 12, 13, 14, 23, 24, 27, 32, 33, 34
Netherlands	**Yes**	**Yes**	**Yes**	**Yes**	**Yes**	Care period can be taken into account when calculating career length requirement for pension and unemployment	**Yes**	**Yes**	**Yes**	4, 6, 8, 12, 13, 14, 15, 19, 21, 22, 23, 24, 26, 27, 30, 31, 32, 33, 34, 35
Ireland	**No**	**Yes**	**Yes** (through carer’s benefit)	**Yes**	N/A	Social insurance coverage/tax credits applied to service vouchers to employ home workers	**Yes**	**Yes**	**yes**	4, 5, 8, 10, 12, 13, 14, 15, 23, 24, 27, 29, 30, 31, 32, 33
*Eastern Europe*										
Bulgaria	**Yes** (possibility to hire spouses)	N/A	**No**	**No**	N/A	N/A	**No**	**Yes**	**No**	4, 8,12, 13, 14, 20, 23, 24, 26, 27, 29, 32, 33
Croatia	**Yes** (for severely disabled care recipients)	**Yes** (cash benefit is limited to spouses/partners below 65 years old)	**No**	**No**	**No**	Social insurance coverage	N/A	**Yes**	N/A	4, 12, 13, 14, 23, 24, 27, 29, 32, 33
Czech Republic	**Yes**	**Yes**	**Yes**	**No**	N/A	Social insurance coverage/ recognition of time spent caring for a close relative as a substitute period for social insurance	**Yes**	**Yes**	**Yes**	3, 4, 6, 7, 8, 10, 12, 13, 14, 20, 23, 24, 26, 27, 29, 30, 31, 32, 33, 35
Estonia	**Yes** (for severely disabled care recipients)	**Yes**	**Yes**	**No**	N/A	Social insurance coverage	**Yes**	**Yes**	**Yes**	4, 11, 12, 13, 14, 22, 23, 24, 26, 27, 29, 31, 32, 33
Hungary	**No**	**Yes**	**No**	**Yes**	N/A	Social insurance coverage	**Yes**	**Yes**	**No**	4, 8, 12, 13, 14, 23, 24, 26, 27, 29, 32, 33
Poland	**Yes** (possibility to hire spouses)	**No**	**Yes**	**No**	N/A	Social insurance coverage	**Yes**	**Yes**	**Yes**	4, 9, 12, 13, 14, 20, 23, 24, 26, 27, 29, 31, 32, 33
Latvia	**Yes**	**No**	**No**	**No**	N/A	N/A	**Yes**	**Yes**	**Yes**	4, 8, 12, 13, 14, 20, 23, 24, 26, 27, 29, 32, 33
Lithuania	**Yes**	**No**	**No**	**Yes**	N/A	Social insurance coverage	**Yes**	**Yes**	**No**	4, 8, 12, 13, 14, 23, 24, 26, 27, 29, 31, 32, 33
Romania	**Yes**	**No**	**Yes**	**No**	N/A	N/A	**Yes**	**Yes**	**No**	4, 8, 12, 13, 14, 23, 24, 26, 27, 29, 32, 33
Slovakia	**Yes**	**Yes**	**Yes**	**No**	N/A	N/A	**No**	**Yes**	**No**	4, 12, 13, 14, 23, 24, 26, 27, 29, 32, 33
Slovenia	**Yes**	**Yes**	**Yes**	**No**	**No**	Social insurance coverage	**Yes**	**Yes**	**Yes**	4, 6, 7, 12, 13, 14, 23, 24, 26, 27, 29, 31, 32, 33
*Southern Europe*										
Cyprus	**Yes**	N/a	**Yes**	**N/a**	**N/A**	Social pension	**Yes** (mainly represented by NGOs or nurses)	**Yes**	**Yes** (mainly represented by NGOs or nurses)	4, 12, 13, 14, 23, 24, 27, 29, 32, 33
Greece	**Yes**	**No**	**No**	**Yes**	N/A	Tax reliefs	**Yes** (mainly represented by NGOs)	**Yes**	**Yes** (mainly represented by NGOs)	1, 4, 8, 9, 12, 13, 14, 23, 24, 27, 29, 32, 33
Italy	**Yes**	**No**	**Yes**	**Yes**	N/A	Pension credits in certain regions; no national policy	**Yes**	**Yes**	**Yes** (mainly represented by NGOs and the voluntary sector)	1, 4, 8, 9, 10, 11, 12, 13, 14, 15, 19, 20, 21, 23, 24, 25, 26, 27, 32, 33
Malta	**Yes**	**No**	**No**	**Yes**	N/A	Pension credits	**Yes**	**Yes**	**Yes**	4, 8, 12, 13, 14, 23, 24, 27, 29, 30, 32, 33
Portugal	**Yes**	**Yes**	**No**	**Yes**	N/A	**NO**	**Yes**	**Yes**	**No**	1, 4, 8, 12, 13, 14, 15, 19, 23, 24, 27, 29, 31, 32, 33
Spain	**Yes**	**Yes**	**Yes**	**Yes**	**No**	Social insurance coverage/exemption from social contributions since 2019/tax credits	**Yes **(mainly represented by secular NGOs and religious charities)	**Yes**	**Yes**	1, 4, 6, 7, 12, 13, 14, 20, 21, 22, 23, 24, 25, 26, 27, 28, 31, 32, 33, 35

Not Applicable (N/A) is indicated when sufficient information is not provided across studies; "Possibility to hire spouses" refers to cash-for-care schemes where the care recipient can use their allowance to formally employ their spouse as a paid caregiver. This contrasts with schemes in some countries (e.g. France) where spouses are explicitly excluded from being remunerated through such benefits

#### Nordic Europe

In Denmark, Finland, and Sweden, the literature (Andersson et al. 2019; Barák 2022; Bouget 2016; Brega et al. 2023; Cahill et al. 2023; Calvó-Perxas et al. 2021; Calvó-Perxas et al. 2018; Castles 2008; Di Rosa 2011; Eggers 2020; Eggers 2023; Esping-Andersen 1990; Esping-Andersen 1999; Eurofound 2025; Ferrera 1996; Jelley et al. 2021; Johansson et al. 2011; Kim et al. 2024; Laporte et al. 2018; Le Bihan 2012; Le Bihan 2019a; Le 2023; Leitner 2003; Lethin et al. 2016; Lister 1994; Lizarondo et al. 2020; McLaughlin 1994) suggests the existence of a comprehensive policy mix to support informal caregivers, including cash benefits for the caregiving dyad, generous paid leave, and social insurance coverage.

In both Denmark and Finland there is a caregiver allowance and the possibility to appoint a carer for home care provision, including informal carers such as spouses. In Denmark, access to paid family care is universal without needs-testing, means-testing, or preconditions regarding the relation to the family carer. In Sweden, the home care allowance involves evaluation of care recipient dependency, with monthly payments based on the level of care required. Municipal employment of caregivers is also accessible as a direct benefit to carers, following assessment in specific circumstances where formal services cannot adequately substitute for family provided care. Overall, the Nordic countries are reported to adopt a universalist approach to eligibility, where access to provision is linked to care needs assessment rather than financial means-testing or the type of relationship with the care recipient. Eligibility criteria for such benefits are usually set at the municipality level. In Denmark and Finland, cash-for-care schemes do not specifically target carers of older adults, though they are eligible.

Social security coverage such as pension credits, health insurance, and unemployment benefits are also provided. For example, in Finland, the kotitalousvähennys tax deduction covers expenses for caring for one's parents, or grandparents, managed by the tax authorities. Additionally, reimbursements are provided for any medical expenses.

Nordic EU countries are also reported to have established care leave schemes. For example, both Sweden and Denmark provide paid care leave for carers of terminally ill relatives (respectively, for up to 100 days, or unlimited duration). In addition to paid care leave, Finland also provides up to 5 days unpaid leave per year for care of relatives requiring immediate care due to serious illness, though there is ongoing debate about making this measure paid.

Public support for respite care is also well-developed, with municipalities primarily responsible for formal services which take precedence over cash-for-care schemes. Sweden offers three types of respite care: home-based services, temporary institutional stays, and alternating care arrangements that rotate between formal facilities and family-based support. The application process requires needs assessment carried out by a care manager, and in some municipalities, in-home respite care is free up to a specific time per month. In Denmark, all citizens have an individual right to public support for extra-familial care without needs-test or means-test. Finland adopts a systematic approach requiring a formal caregiver contract, guaranteeing at least two days of monthly respite, depending on care intensity. Additional respite care services in the Nordic countries include rehabilitation, innovative assistive technology and—in some cases—night care centres, to promote independent living for the older adults and provide relief for carers. Macro-level indicators confirm that Nordic countries rank high in public provision; Sweden has one of Europe’s highest availabilities of LTC beds, while Denmark and Finland maintain high numbers of LTC workers per older person. Overall, comparative evidence highlights that Nordic LTC systems are generous and universal, with a central role for home care. However, their decentralised governance creates regional variations in access and service levels.

Finally, interventions targeting caregivers’ training and well-being are also established although are reported to be less accessible and to vary across municipalities. In Denmark, the government subsidises services like support groups, educational programmes, and psychological counselling. Finland provides support networks and social counselling and implements voluntary ‘caregivers health checks’ for those with at least two years of experience to monitor their health. In Sweden, caregivers receive social and emotional support through groups and counselling, though quality can vary. Municipal family consultants offer free counselling and referrals without a needs assessment, and the healthcare sector provides group courses for specific conditions like dementia.

#### Continental Europe

Continental countries like Austria, Belgium, France, Germany, Luxembourg, and the Netherlands (Barák 2022; Brega et al. 2023; Cahill 2023; Calvó-Perxas et al. 2021; Calvó-Perxas 2018; Castles 2008; Courtin et al. 2014; Di Rosa et al. 2011; Eggers 2020; Eggers 2023; Esping-Andersen 1990; European Commission 2021; Eurostat 2024; Ferrera 1996; Genet et al. 2012; Jelley et al. 2021; Johansson et al. 2011; Kim et al. 2024; Laporte Uribe et al. 2018; Le Bihan 2012; Le Bihan 2019a; Le 2023; Leitner 2003; Lethin et al. 2016; Lister 1994; Lizarondo 2020; Llena-Nozal 2025; McLaughlin 1994) provide various forms of support, including cash benefits and labour market policies. According to macro-level indicators, these systems score in the medium-to-high range on caregiver support, reflecting the availability of allowances, credits, and leave arrangements. They are characterised by integrated policy frameworks combining employment rights and LTC benefits, though generosity and accessibility vary between and within countries.

Belgium, Germany, and the Netherlands provide comprehensive cash-for-care schemes for both members of the care dyad. In Austria, Belgium, France, Germany, and Luxembourg, cash benefits for care recipients often compensate family caregivers, although France prohibits the remuneration of spouses. The generosity of these schemes can differ substantially within a country; for instance, while Belgium offers comprehensive support, financial aid varies by region, with some municipal allowances as low as €50 per month. Eligibility criteria also vary across countries. France, Germany, and the Netherlands require a formal contract or health plan with the caregiver. While the Netherlands sets no minimum dependency level, Austria and Germany categorise care needs into different levels. France’s APA benefit (Allocation Personnalisée d'Autonomie) is restricted to care recipients aged 60 and over.

Social insurance coverage policies in continental countries vary, often including pension credits, health insurance, and unemployment benefits. Germany provides pension entitlements to those performing significant care (over ten hours per week) while working limited hours in formal employment. France allows caregivers to validate full pension quarters for care-related career interruptions through its 2023 old-age insurance policy and, like Belgium, uses tax credits in the form of service vouchers to employ home workers. In the Netherlands, care periods can count towards pension and unemployment calculations, and municipalities offer free volunteer insurance to unpaid carers. Austria's comprehensive approach provides pension and health protection through formal employment relationships for caregivers, a policy expanded in 2024 to include non-family members. Similarly, Luxembourg's dependency insurance covers pension contributions up to the minimum social wage for caregivers who meet specific conditions.

Several continental EU countries have implemented paid care leave schemes. In Belgium, thematic leave allows employees to suspend work for up to three months for palliative care or up to twelve months for medical assistance to a seriously ill family member. Austria’s 'Pflegekarenz' provides up to three months of paid leave for the care of close relatives receiving an allowance. Germany offers several options: paid leave; up to six months of job-protected unpaid leave with social insurance coverage; and the right to reduce work to a minimum of 15 h per week for up to two years. In France, caregivers can take up to three months of leave, including for end-of-life care, though it is reported as largely unpaid with low compensation when provided (e.g. €43/day). Several countries, including Austria, Belgium, France, and Germany, and the Netherlands, also offer flexible work arrangements, such as the right to reduce weekly working hours or adjust schedules—to help caregivers facilitate the work-care balance.

The literature indicates that continental EU countries adopt a mixed LTC policy focused on home and community care, with services typically organised at regional and municipal levels. Respite care is supported through various means; for instance, Austria provides annual care grants ranging from €1200 to €2200 for up to 28 days, with additional support for carers of people with dementia, while Germany’s LTC insurance offers cash benefits and the right to four weeks of respite care per year. Social care, including assistance with daily activities like cleaning and cooking, is available in Belgium, Germany, and Luxembourg. Furthermore, France, Germany, and Luxembourg offer prevention and rehabilitation services, nursing aids, and home adaptations. Macro-level indicators reveal significant variation in public provision. The Netherlands and Luxembourg have among the highest levels of public provision in Europe, measured by LTC bed availability per 1000 older people. LTC worker availability is also highest in Luxembourg and the Netherlands, while it remains lower in Austria, Belgium, and France. Public funding generosity differs as well: Luxembourg and the Netherlands cover the vast majority of LTC costs, and Austria and Belgium also have high public funding coverage. France, however, covers substantially less, resulting in high out-of-pocket expenses.

At the same time, comparative evidence shows that decentralised governance creates regional disparities. In countries such as Austria, Belgium, and Germany, the division of responsibilities between regional and local authorities leads to variation in access to home care and other respite services. Belgium has strongly regionalised systems, with Flanders, Wallonia, and Brussels each managing their own LTC arrangements. Germany’s national LTC insurance is implemented locally through insurers and municipalities, creating differences at the local level. In Austria, responsibilities are divided between Länder and municipalities, also leading to variation in access. In the Netherlands, the decentralisation of home support to municipalities under the Social Support Act (WMO), including temporary respite care for informal caregivers, has similarly raised concerns about unequal access. In contrast, Luxembourg’s LTC insurance appears to be more centrally organised.

Finally, the Continental countries offer measures aimed at caregiver well-being, including training, information, and psychological support. However, the focus of in-kind benefits in these countries is primarily on the care recipient, with fewer services directly targeting caregivers compared to the Nordic systems. In Germany, caregivers benefit from a system that includes counselling and training interventions, online support services, a dedicated hotline for unpaid caregivers in critical situations, and the ‘Wege zur Pflege’ government web portal, which provides information on the care system. In Austria, organisations such as Caritas offer structured courses covering nursing activities and fall prevention, often subsidised by the government. The Netherlands provides counselling and information services, funded by municipalities, who are available to all unpaid carers to provide information, advice, and help with care service applications. Furthermore, several countries provide targeted support for specific care groups, such as young carers. Germany’s ‘Pausentaste’ initiative offers online platforms, hotlines, and counselling services designed for young carers. The Netherlands established the Strategic Alliance for Young Carers in 2020, which acts as a network for young carers under 25 and professionals to facilitate knowledge exchange and connection. Lastly, formalised assessment of caregivers' needs is also provided in Austria, Germany, and France.

#### Ireland

In Ireland (Barák et al. 2022; Bouget et al. 2016; Calvó-Perxas et al. 2021; Castles 2008; Di Rosa et al. 2011; Eggers et al. 2020; Eggers 2023; Esping-Andersen 1990; Johansson et al. 2011; Kim et al. 2024; Le Bihan et al. 2019a; Le 2023; Leitner 2003; Lethin et al. 2016; Lister 1994; Lizarondo et al. 2020), support primarily consists of means-tested direct payments to co-resident caregivers, with eligibility criteria based on the care recipient’s dependency, the carer’s employment record, and proof of full-time caregiving. Additional financial support includes the “home carer's tax credit” for jointly assessed married couples or civil partners where one partner provides care at home.

In terms of leave, paid and unpaid carer leave is offered, which can also be used for non-family members. Home care services, providing respite and non-medical assistance, are delivered by community organisations or private agencies but lack statutory regulation, resulting to a significant under-provision of public LTC services. This is consistent with macro-level indicators that position Ireland higher on caregiver support but lower on service-based provision.

Since 2017, minor reforms funded by dormant accounts have introduced resources like supportive online apps and self-help networks. However, training and counselling are often delivered by voluntary organisations with public grant support. Furthermore, since 2018, all those receiving a caregiver allowance are eligible for general practitioner cards, providing them with free GP services to help maintain their own health.

#### Eastern Europe

Literature on Eastern European countries—including Bulgaria, Czech Republic, Croatia, Estonia, Hungary, Latvia, Lithuania, Poland, Romania, Slovakia, and Slovenia (Arts 2002; Barák et al. 2022; Brega et al. 2023; Cahill 2023; Calvó-Perxas et al. 2021; Calvó-Perxas et al. 2018; Castles 2008; Courtin 2014; Di Rosa et al. 2011; Eggers et al. 2020; Eggers 2023; Ferrera 1996; Jelley et al. 2021; Johansson 2011; Kim 2024; Le Bihan 2012; Le Bihan 2019a; Le 2023; Leitner 2003; Lethin et al. 2016; Lister 1994; Lizarondo et al. 2020; McLaughlin 1994)—is relatively limited. Consistent with macro-level indicators, most countries in this region score at the lower end of caregiver support and public provision.

In some countries, such as Hungary, Latvia, and Lithuania, family caregiving responsibilities are legally mandated and sometimes enshrined in the country’s Constitution. In Estonia, the Family Law Act (Perekonnaseadus) requires adult relatives up to the second degree of kinship to provide care.

Among the Eastern countries with cash-for-care policies, eligibility criteria vary significantly based on factors such as the care recipient’s age, dependency level, household income, and the caregiver’s relationship to the recipient. Slovakia and Slovenia offer both types of cash benefits; in Slovakia the caregiver allowance is means-tested, depending on the severity of the recipient’s condition and whether they co-reside with the caregiver; it is also unavailable if the care recipient already receives a care allowance. In Slovenia, caregivers who leave employment for care duties receive a monthly payment for lost income, along with social security and healthcare rights. A recent requirement under its 2023 Long-Term Care Act mandates that these carers complete 30 h of initial training and 20 h of refresher training every three years. Hungary provides cash benefits exclusively for caregivers of relatives with disabilities or permanent illnesses. Bulgaria, Latvia, and Romania offer benefits for care recipients with severe disabilities. Similarly, Bulgaria offers a structured employment scheme allowing individuals, including family members, to provide paid assistance to older adults and people with disabilities. The Czech Republic provides two additional allowances for people with disabilities: the mobility allowance and the special-aid allowance. In Estonia, most family carers are unable to receive cash benefits (Hooldajatoetus) from local authorities, and when granted, the extent of pay is very low. These variations align with indicator findings that Eastern systems rely more on targeted cash support for recipients than on broad-based allowances for caregivers.

Our review reveals significant disparities for leave care schemes in Eastern Europe. Long-term paid care leave schemes appear to be largely absent in Bulgaria, Croatia, Latvia, and Lithuania, where support is often limited to short-term entitlements. In contrast, both the Czech Republic and Slovakia offer extensive paid leave, providing up to 90 days of wage replacement at 60% and 55% of earnings, respectively, for those caring for seriously ill relatives. Following recent EU directives, Romania provides 5 days of paid carer's leave annually. Hungary grants up to two years of unpaid leave for caregivers of permanently ill relatives. Estonia provides only a temporary benefit (Hooldushüvitis) for short-term leave in case of emergencies.

While data on social insurance coverage are missing for Bulgaria and Romania, literature indicates that the Czech Republic, Poland, Lithuania, Slovenia do provide such benefits. For example, caregivers in Poland receive health and unemployment insurance. Czech Republic also recognises time spent caring for a close relative as a substitute period for social insurance, contributing to pension rights since 2007.

Further, respite care services are reported to be available across all Eastern European countries, though their type and intensity vary significantly, not only between nations but also at regional and municipal levels. Specific national frameworks illustrate this diversity. For instance, Lithuania's respite care is capped at 720 h annually per family, though extensions are possible in exceptional circumstances through a co-funding mechanism. Poland differentiates its provision, offering a maximum of 240 h for day respite and 14 days for 24-h care. Meanwhile, Slovakia permits up to 30 days of respite care per year. Comparative research highlights that the formal home and community care sectors in several Eastern European countries remain underdeveloped, leading to significant inequalities in access and service coverage. These services are often restrictive, primarily targeting care recipients with severe disabilities or high degrees of dependency. An example is Estonia, where national legislation obliges municipalities to provide basic care services, but coverage is often inadequate due to variation between regions based on local government budgetary resources, with older persons typically eligible only if their children do not live in the same municipality. Macro-level indicators further position Eastern Europe at the lower end of service-based provision. The availability of LTC beds, for example, lags behind other regions, with the number of beds per older person being particularly low in Latvia. The availability of LTC workers is also low in Latvia and Hungary, although Estonia reports a relatively high number of workers per older person. Public funding for these services varies considerably. Hungary, for instance, covers a relatively high share of LTC costs. In contrast, Estonia covers very little, resulting in high out-of-pocket expenses for care recipients and their families.

Finally, data on the availability of psychological support and training for caregivers in many Eastern European countries are notably scarce. This reflects a broader trend where such in-kind benefits are largely underdeveloped across the region. Rather than being statutory entitlements, available support is typically delivered through local NGOs or municipalities. Consequently, these programmes often depend on external, project-based funding, making them less stable. For example, in Poland, the Wola district municipality of Warsaw collaborates with various NGOs to organise support programmes for informal and young carers, including lectures by specialists and individual consultations with psychologists.

#### Southern Europe

In Cyprus, Greece, Italy, Malta, Portugal, and Spain (Albuquerque 2022; Barák 2022; Brega et al. 2023; Cahill et al. 2023; Calvó-Perxas et al. 2021; Calvó-Perxas et al. 2018; Castles 2008; Courtin 2014; Di Rosa 2011; Eggers 2020; Eggers 2023; Esping-Andersen 1990; Eurostat 2024; Ferrera 1996; Genet et al. 2012; Jelley et al. 2021; Johansson et al. 2011; Kim et al. 2024; Laporte Uribe et al. 2018; Le Bihan 2012; Le Bihan 2019a; Le Bihan et al. 2019b; Le 2023; Leitner 2003; Lethin et al. 2016; Lister 1994; Lizarondo 2020; McLaughlin 1994), policies for caregivers are generally reported to be more limited. Additionally, little data are available for Cyprus, Greece, and Malta, where reports indicate a lack of direct cash benefits for caregivers and limited provisions of ad-hoc labour market policies. This corresponds with macro-level indicators, which place Southern European countries at the lower end of caregiver support compared to other EU regions.

Cash-for-care schemes in Southern Europe vary, though traditionally such benefits are provided to the dependent person rather than directly to the caregiver, with eligibility commonly depending on certified disability or high dependency rather than income alone. Spain provides both indirect and direct benefits, although their generosity is reportedly low and, in some regions, varies with the beneficiary's economic condition. Portugal also offers cash benefits, requiring caregivers who apply for its “Support allowance for the main informal caregiver” to provide permanent care and be officially recognised by social security services, a process which requires the care recipient to explicitly confirm their choice. In both Spain and Portugal, co-residence is a prerequisite and means-testing is applied to both the caregiver and the care recipient. While non-co-resident caregivers in Portugal are ineligible for the allowance, they can still access other supports like respite care. In Italy, cash benefits are primarily directed towards the care recipient, with the main LTC scheme being the “companion allowance” for individuals with severe disabilities, without age or income restrictions. This is a flat-rate monthly payment, and access requires a certification of 100% disability, with the overall generosity of this support for family care to be thus considered low. In Greece and Cyprus, financial support for care recipients is provided through social security schemes and professional funds. In Malta, the cash benefit is only available to older recipients on the residential care waiting list.

Regarding additional benefits, Spain has introduced a tax deduction for those combining paid employment with caregiving responsibilities. Similarly, in Greece, caregivers can benefit indirectly from some income tax relief when supporting a relative with disabilities. Italy allows for the possibility of earlier retirement for co-resident caregivers, under specific regional schemes; however, the main cash benefit scheme, “companion allowance”, provides no social security coverage for the caregiver. Malta and Cyprus are reported to provide pension credits to caregivers who leave the workforce to provide care.

Care leave schemes vary considerably among Southern countries. Spain offers a range of options, including paid leave for up to three months and unpaid LTC leave of up to two years, although the use of paid leave is reported to be limited. Paid care leave has existed in Italy since the 1990s. Italy provides three paid days per month or two years of paid leave for care of severely disabled family members or coresidents. Cyprus introduced “The Leave and Flexible Working Arrangements for Work-Life Balance Law 2022” providing five days of paid leave per year for caregivers. Malta provides unpaid leave, including urgent family leave and responsibility leave for public sector employees. Greece and Portugal offer statutory rights to unpaid leave for significant family responsibilities, such as caring for an older parent with LTC needs.

While respite care services exist across Southern Europe, they are often reported to be underdeveloped, with home and residential care typically targeting older adults with high dependency needs. In Spain, day care centres provide essential respite for caregivers, usually from 9:00 to 17:00, five days a week, but regional governments set their own rules for user contributions and service charges. This variability on eligibility and co-payments leads to unequal access depending on where people live. Similarly, in Italy, devolved regional responsibilities have resulted in a north–south divide, with northern regions generally better resourced. As minimum standards are not legally defined, eligibility criteria for needs-testing differ strongly, though home care is fully funded for 30 days after a hospital discharge. In Portugal, Greece, Cyprus, and Malta, respite and home care are described as more limited and fragmented, with municipalities and NGOs playing a strong role due to weaker formal public structures. Greece's “Help-at-home” programme has strict eligibility based on age, income, and health, primarily targeting people in economic hardship without support. The substantial gap between demand and supply in many Southern countries has fostered a large private sector for those who can afford care. Macro-level indicators confirm this trend, showing Southern Europe has some of the lowest levels of public provision. The number of LTC beds is particularly low in Italy, Greece, and Portugal, and LTC worker availability is very low in Greece, while Spain maintains higher levels. Public funding is also limited: Italy and Spain cover around half of LTC costs, Greece less than half, and Portugal less than a quarter, often resulting in high out-of-pocket expenses.

Finally, training, information, and counselling for caregivers are reported to rely heavily on the voluntary sector, including NGOs, religious organisations, and academic institutions. For example, the Cyprus University of Technology conducts workshops specifically for unpaid caregivers of individuals with dementia. Italy shows some initial recognition of young carers providing support through “Initiatives for young carers in schools and universities” which offer more flexible schedules and skills development opportunities to help them balance caregiving with education.

## Discussion

This systematic review synthesises evidence from 35 studies on LTC policies supporting informal caregivers of older adults across the EU. The analysis reveals several key findings. First, significant geographical imbalances in research coverage; second, substantial variation in both the types and comprehensiveness of policy instruments adopted; and third, patterns of cross-national variation that generally align with welfare regime typologies, though with important within-regional heterogeneities that challenge simple categorisations.

### International research coverage

The research coverage and the level of analysis for each country are highly uneven. While broad cross-national reports ensure some representation for most EU member states, the intensity of dedicated national-level analysis differs markedly. Many studies and policy information are available for countries such as Germany, France, Sweden, Spain, and the Netherlands, while fewer articles or less in-depth information covers countries such as Bulgaria, Croatia, Cyprus, Hungary, Romania, Malta, and Slovakia, which are often only briefly mentioned in multi-country, international reports, rather than being the focus of stand-alone investigations. This scarcity makes it difficult to update information for the less-documented countries. Thus, some of the missing/incomplete data may not reflect an actual gap in the policy instruments adopted by national governments but rather a lack of international work on the topic. This research imbalance has implications beyond data availability—it shapes policy learning and implementation patterns, as countries with limited international documentation may be excluded from comparative policy analyses and cross-national learning opportunities. The concentration of information Nordic and Continental EU countries may therefore reinforce existing knowledge hierarchies and limit understanding of policy innovation in less-studied contexts. This observation may be particularly relevant for EU-funded programmes like Horizon Europe. Relying on data from a subset of member states might risk overlooking the specific institutional realities of certain Southern and Eastern EU countries. Future research could therefore benefit from going beyond mere representation in datasets, potentially through strategies that support local capacity building and encourage leadership from less-studied regions.

### LTC policy adoption and comprehensiveness

Examining policy comprehensiveness reveals distinct patterns across the EU. Of the nine categories of instruments considered (see Table 2), Belgium and the Netherlands are reported to adopt instruments that fall into all of them. Austria, Finland, Germany, and Spain adopt eight out of the nine types of instruments, featuring more comprehensive approaches that address multiple caregivers' needs. Denmark and Sweden appear to adopt seven out of the nine categories, though they appear to prioritise paid work leave, public service provision, and social insurance coverage over direct cash allowances. Conversely, countries such as Bulgaria, Romania, and Slovakia rely on only two or four types of instruments, suggesting a narrower policy mix. Across the EU, cash (direct or indirect) allowances and in-kind respite care services emerge as the most widely used policy instruments, reported across all 27 EU states. This near “universal” adoption of cash-for-care schemes and respite care services is noteworthy, as it occurs across all welfare regime types despite different theoretical orientations towards caregiving.

Yet significant heterogeneities exist in how these instruments are implemented. Comprehensiveness in formal policy adoption does not necessarily translate to effective or accessible support. The literature reveals substantial variation in policy generosity, eligibility criteria, and implementation even where instruments formally exist.

Despite their near-universal adoption, cash-for-care schemes show substantial variation in design and logic across Europe. A primary distinction is who receives the benefit: Nordic and some Continental systems often support both the caregiver and recipient, whereas Southern and Eastern European models traditionally direct funds to the care recipient, and Ireland focuses exclusively on the caregiver. Eligibility criteria also diverge, contrasting the universalist, needs-based approach of Nordic countries with the complex rules in Continental systems, which can include specific dependency levels or prohibitions on spousal payments. Generosity is also a key differentiator; it is generally reported to be low in Southern and Eastern Europe, while decentralisation in some Continental countries can also undermine national policy commitments, resulting in significant regional differences in the actual support.

The provision of respite care varies significantly across EU, reflecting fundamental differences in policy adoption and comprehensiveness. The Nordic followed by the Continental countries have more developed systems, though they are organised differently. Nordic models are characterised by more universal—even though municipally-led—formal services that take precedence over cash schemes. Continental systems are defined by more decentralised governance, where a mix of national insurance frameworks, regional grants, and local implementation leads to significant internal variation in access and generosity. Provision in Southern and Eastern Europe is far more fragmented and underdeveloped, often targeting only those with the highest dependency needs. In these regions, weaker formal public structures mean that municipalities, NGOs, and the voluntary sector play a much stronger role in service delivery, which creates sharp territorial inequalities and fuels a large private market to fill the gaps.

The availability and design of care leave schemes reveal a clear divide across European welfare states. The Nordic and Continental countries provide the most generous and established systems, characterised by paid, long-duration leave, particularly for palliative or end-of-life care. In contrast, Eastern Europe presents a landscape of stark disparities, with some nations (e.g. Czech Republic and Slovakia) offering paid leave that rivals Continental provisions, while others report no long-term schemes at all. The situation in Southern Europe is also mixed; while a few countries have established paid leave, especially for severe disability cases, others rely solely on unpaid leave options. Further, flexible work arrangements for caregivers remain a notably underdeveloped policy area, reported more frequently in Continental countries, which highlights a significant gap in work-life balance policies even within the most supportive welfare systems. Countries such as the Netherlands provide flexible work arrangements universally, resulting in wider access, although research suggests that gender imbalances remain (Brega et al. 2023). In contrast, many others provide either limited support or do not report established schemes for flexible work.

The provision of social security coverage for caregivers also reveals divides across EU welfare states. Continental and Nordic countries are reported to offer more formalised systems, where caregiving periods are recognised for pension calculations and other insurance benefits. In particular, continental systems demonstrate structural innovation by formalising caregiver employment, validating pension quarters, and using dependency insurance to cover contributions. In contrast, Eastern Europe presents a mixed landscape, with some countries offering coverage, while others report no provisions or lack available data entirely. Limited support is also found in Southern Europe, where national policies, with the exception of Spain, are often absent, and coverage is fragmented, relying on minimal tax relief, regional-level pension credits, or is simply not provided.

Support for caregiver well-being, such as training and psychological support, shows structural differences across EU. Both Nordic and Continental countries have state-supported systems, but their focus differs. Nordic models are often more caregiver-centric in their design, directly targeting caregiver needs, though the accessibility and quality of these services can vary by municipality. In contrast, Continental systems are often more formalised, incorporating features like needs assessments for caregivers, even if the primary focus of in-kind benefits remains on the care recipient. This contrasts with Southern and Eastern EU countries, where provision is significantly less developed and institutionalised. In these regions, support is fragmented and relies heavily on a network of non-state actors, including the voluntary sector and NGOs, often sustained by unstable, project-based funding rather than statutory entitlement. Ireland reflects a similar dependence on voluntary organisations but stands out for providing a direct health benefit to caregivers. Across all regimes, tailored support for distinct groups, such as young carers, is an emerging but not yet consistently integrated policy area.

### Cross-national variation in EU long-term care policies

Cross-national variations in EU care policy, as described above, align with welfare regime typologies, supporting established theoretical frameworks while also revealing important nuances. Following foundational work (Leitner 2003; Saraceno and Keck 2011) and more recent analyses (e.g. Eggers et al. 2020, 2023; Le Bihan et al. 2019), the findings show that policy logics diverge based on how states operationalise care responsibility and the boundaries between public and family obligations.

Nordic countries demonstrate a “defamilialisation” orientation, prioritising public provision and in-kind services (Leitner 2003). This model is built on the principle of universalism, where the state assumes primary responsibility for care, framing it as a social right. This orientation is also seen in our findings, which show that formal services and respite care are legally mandated, universally accessible, and take precedence over cash schemes, thereby granting citizens autonomy from family dependencies. However, the findings on the establishment and evolution of caregiver allowances, municipal employment schemes, and generous social insurance also reflect a significant policy development. These specific instruments align with what Le Bihan et al. (2019) identify as a turn towards “optional familialism” even within traditionally defamilialised states. This trend does not replace the core defamilialised logic but supplements it; by providing direct support that remunerates family care, the state increasingly validates and empowers it as a viable choice alongside its public services.

Continental countries exemplify “supported familialism”, where family care is recognised as socially valuable work deserving of multifaceted support. The policy mix in this regime is designed to enable and sustain care within the family, aligning with the conservative-corporatist principle of subsidiarity (Saraceno and Keck 2011). The findings demonstrate a high density of support instruments—including comprehensive cash-for-care schemes, paid leave, and extensive social insurance coverage—which shows an institutional commitment to constructing care provision as a legitimate alternative to formal services. This approach is the clearest expression of what Le Bihan et al. (2019) term “optional familialism”, where the state actively empowers families to provide care through formalised support. The review's findings on the formalisation of caregiver employment in Austria, the validation of pension quarters in France, and dependency insurance covering contributions in Luxembourg are examples of this logic. Further, the combination of more developed public services with more generous support for family care compared to Eastern and Southern EU, may exemplify the argument from Eggers et al. (2020) that high levels of both de-familialising and familialising policies can coexist within a single system. A key function of this model is its capacity to mitigate the associated risks for caregivers, such as loss of income and pension gaps, thereby “professionalising” and validating their role in a way that is distinct from the more service-oriented Nordic model (Eggers et al. 2023).

Southern and Eastern EU countries largely exhibit “familialism by default”, where families bear primary care responsibility amidst limited public alternatives and systematic support (Saraceno and Keck 2011). The findings confirm that support in these regions is fragmented, with underdeveloped public services and a reliance on cash benefits that are often low in generosity and targeted at the care recipient based on high dependency needs. Framed within the conceptualisation of Eggers et al. (2020), these countries are characterised by lower levels of generosity in both de-familialising and familialising policy dimensions. This policy structure is associated with high social risks for caregivers, who often face significant financial penalties and inadequate social security protection (Eggers et al. 2023). Further, as Le Bihan et al. (2019) argue, the cash-for-care schemes in this context, absent a strong public service infrastructure, can inadvertently foster the growth of a low-cost, unregulated care market, which becomes the default solution for families.

### Regional heterogeneities and disparities

While broad welfare typologies are confirmed by our findings, the review also reveals significant heterogeneities that challenge homogeneous classifications, particularly within the Southern and Eastern European regimes. In Southern Europe, the findings show that Spain demonstrates a policy mix more aligned with the Continental approach, especially regarding social insurance for caregivers. This is consistent with analyses by Eggers et al. (2023) that highlight Spain’s stronger social security rights for carers and supports classic observations that Southern welfare states show significant internal variability (Ferrera 1996). Similarly, within Eastern Europe, the findings suggest that countries like the Czech Republic offer stronger support than regional patterns would suggest. This indicates the existence of diverse post-socialist welfare trajectories rather than a uniform model of underdevelopment, reflecting different paths of policy learning and adaptation since their integration into the EU.

Beyond these cross-national variations, the findings indicate that significant intra-national disparities, often created by decentralisation (Albuquerque 2022; Genet et al. 2012; Llena-Nozal et al. 2025; Riedel et al. 2016), affect all welfare regime types. In Continental countries, decentralisation is reported to create variation despite comprehensive national frameworks Belgium's regionalised systems, Germany's local implementation, and the Netherlands' municipal responsibilities all raise concerns about unequal access. This aligns with comparative evidence documenting that the decentralisation of LTC governance creates regional inequalities in service access and generosity, even in otherwise well-resourced systems (Pavolini & Ranci 2008). In Nordic countries, despite high overall provision, the findings show that strong municipal autonomy creates local variation in the accessibility of training and support services. Territorial inequalities are particularly sharp in Southern countries, as evidenced by Italy's north–south divide and the geographical disparities created by Spain's autonomous communities. These regional patterns confirm that an individual’s place of residence is a key determinant of support accessibility, and that national policy frameworks may mask substantial territorial inequalities across all regions.

### Implications for policy

The current review suggests a significant heterogeneity in policy support for informal caregivers across the EU, creating an uneven landscape for mitigating the associated social risks. Caregivers face well-documented risks including career interruptions, subsequent financial insecurity, and diminished well-being (Eurostat 2024; Verbakel 2014). These risks are disproportionately borne by women, who constitute the majority of caregivers and are therefore more exposed to the long-term consequences of policy gaps (Le & Ibuka 2023; Quashie et al. 2022). Our review identified wide disparities in financial support, paid leave and social security, and uneven access to services like respite care, caregiver training, and psychological support. These policy variations translate to varying levels of protection against income loss, old-age insecurity, and the mental and physical strain of caregiving. From these findings, several key policy implications emerge.

The necessity for a more integrated policy approach is clear. To prevent the social and economic costs of care from falling heavily on caregivers, a comprehensive safety net is required, where financial supports, labour market integration measures, and direct services are systematically combined. Further, the policy variations identified, particularly the divide between Northern/Continental and Southern/Eastern Europe, highlight the need for greater policy convergence and knowledge transfer. Encouraging the adoption of successful policy models from countries with more comprehensive systems could help address clear gaps in regions with less developed frameworks. Finally, the findings point to the importance of implementing formal needs assessments for caregivers across the EU. The diversity of support measures suggests that a one-size-fits-all approach is inadequate. By systematically assessing the individual needs of caregivers, considering their financial situation, health, and the intensity of their care duties, EU policies can be better tailored, ensuring that support is allocated more effectively and efficiently to those who require it.

### Limitations

Despite the insights provided by this review, several limitations must be noted. First, the review reflects what has been reported in the existing literature, rather than providing an inventory of policies. While this approach allows for a synthesis of how policies are framed and evaluated, it means our findings are contingent on the scope and coverage of the published evidence, therefore complementing policy documents. This leads to two related limitations: a language bias, as restricting the review to English-language publications may have oversampled studies from Nordic and Continental Europe; and a resulting coverage bias, where certain Southern and Eastern European countries may appear underrepresented due to a lack of research published in English, not necessarily an absence of policies. For instance, during the screening process, we estimated that language constraints were the primary exclusion criterion for more than 17 potentially relevant records, suggesting that specific national-level insights were likely missed. Additionally, due to these constraints, a substantial portion of non-English grey literature could not be assessed. Therefore, the strategy likely under-reports local-level policies, particularly those implemented with NGOs, as they are often documented in national-language reports rather than international academic journals.

In addition, the imbalances identified in the review, pertaining also to the international research coverage, may partly reflect the systematic biases inherent in the comparative databases often used as primary sources in the literature. Established datasets such as the Mutual Information System on Social Protection (MISSOC), while invaluable, often face challenges regarding data homogeneity, comparability, and granularity. Consequently, comparative studies and reports used in the current review may inadvertently reproduce some of these gaps. Future research could mitigate this by adopting more rigorous, systematic mapping methodologies to develop more comprehensive LTC policy databases, which triangulate secondary literature with primary source verification to ensure a more accurate representation of national policy landscapes across the EU (Viero & Fischer 2025).

Furthermore, the binary classification of the different policy instruments (Table 2), while enabling cross-national comparison of policy adoption, does not capture variations in policy generosity, eligibility restrictions, or implementation quality—dimensions that although are discussed in our findings, require more detailed country-by-country analysis beyond the nature of a systematic review. Finally, the review's scope was intentionally limited to EU member states, meaning it does not cover non-EU countries like Norway or the UK, which could offer valuable comparative insights. Future research should aim to close these knowledge gaps by replicating similar analyses with multilingual search strategies to systematically include non-English sources, thus providing a more comprehensive understanding.

## Conclusion

This systematic review suggests significant variations in care support policies across the EU, with more comprehensive systems typically found in Nordic and Continental countries compared to more limited frameworks in Southern and Eastern EU. This disparity, alongside substantial heterogeneities within nations often driven by decentralisation, highlights the clear potential for more targeted policy transfer and cross-national learning. To address the growing demand for care as Europe’s population ages, governments must adopt a multidimensional approach that strengthens support systems by integrating comprehensive financial assistance and social security, paid care leave schemes, and in-kind services including respite care, training, and psychological support. With caregiving forming the backbone of LTC systems, addressing these policy gaps is not only a matter of social justice but is also critical for the sustainability of effective care. By investing in care support, EU countries can build more resilient LTC systems that enhance both care quality and the socio-economic security of caregivers.

## Data Availability

Data sharing is not applicable to this article as no datasets were generated or analysed during the current study.
